# Mobile Device Ownership, Current Use, and Interest in Mobile Health Interventions Among Low-Income Older Chinese Immigrants With Type 2 Diabetes: Cross-sectional Survey Study

**DOI:** 10.2196/27355

**Published:** 2022-02-02

**Authors:** Lu Hu, Chau Trinh-Shevrin, Nadia Islam, Bei Wu, Shimin Cao, Jincong Freeman, Mary Ann Sevick

**Affiliations:** 1 Center for Healthful Behavior Change Department of Population Health, New York University Grossman School of Medicine New York University Langone Health New York, NY United States; 2 Department of Population Health New York University Grossman School of Medicine New York University Langone Health New York, NY United States; 3 Rory Meyers College of Nursing New York University New York, NY United States; 4 Charles B Wang Community Health Center New York, NY United States; 5 Milken Institute School of Public Health George Washington University Washington, DC United States

**Keywords:** technology use, Chinese immigrants, type 2 diabetes, mHealth, health disparities, immigrant health, diabetes, mobile health, intervention, smartphone, immigrant

## Abstract

**Background:**

Chinese immigrants suffer a disproportionately high type 2 diabetes (T2D) burden and tend to have poorly controlled disease. Mobile health (mHealth) interventions have been shown to increase access to care and improve chronic disease management in minority populations. However, such interventions have not been developed for or tested in Chinese immigrants with T2D.

**Objective:**

This study aims to examine mobile device ownership, current use, and interest in mHealth interventions among Chinese immigrants with T2D.

**Methods:**

In a cross-sectional survey, Chinese immigrants with T2D were recruited from Chinese community centers in New York City. Sociodemographic characteristics, mobile device ownership, current use of social media software applications, current use of technology for health-related purposes, and interest in using mHealth for T2D management were assessed. Surveys were administered face-to-face by bilingual study staff in the participant’s preferred language. Descriptive statistics were used to characterize the study sample and summarize technology use.

**Results:**

The sample (N=91) was predominantly female (n=57, 63%), married (n=68, 75%), and had a high school education or less (n=58, 64%); most participants had an annual household income of less than US $25,000 (n=63, 69%) and had limited English proficiency (n=78, 86%). The sample had a mean age of 70 (SD 11) years. Almost all (90/91, 99%) participants had a mobile device (eg, basic cell phones, smart devices), and the majority (n=83, 91%) reported owning a smart device (eg, smartphone or tablet). WeChat was the most commonly used social media platform (65/91, 71%). When asked about their top source for diabetes-related information, 63 of the 91 participants (69%) reported health care providers, followed by 13 who reported the internet (14%), and 10 who reported family, friends, and coworkers (11%). Less than one-quarter (21/91, 23%) of the sample reported using the internet to search for diabetes-related information in the past 12 months. About one-third of the sample (34/91, 37%) reported that they had watched a health-related video on their cell phone or computer in the past 12 months. The majority (69/91, 76%) of participants reported interest in receiving an mHealth intervention in the future to help with T2D management.

**Conclusions:**

Despite high mobile device ownership, the current use of technology for health-related issues remained low in older Chinese immigrants with T2D. Given the strong interest in future mHealth interventions and high levels of social media use (eg, WeChat), future studies should consider how to leverage these existing low-cost platforms and deliver tailored mHealth interventions to this fast-growing minority group.

## Introduction

Type 2 diabetes (T2D) in Chinese Americans is a significant health concern for the US health care system [1-3]. According to a recent epidemiological survey, 43.1%, or 1 out of every 2, Chinese Americans in New York City (NYC) have T2D or prediabetes [3]. The majority of Chinese Americans with T2D are foreign-born older immigrants with limited English proficiency and health literacy [3-6].

Once diagnosed with T2D, Chinese immigrants demonstrate poorer self-management and worse glycemic control [4,5,7-9], and are more likely to develop end-stage renal disease [7,10]. In a recent study of racial and ethnic minorities with T2D in NYC [5], Chinese immigrants performed an average of 3.80 (SD 0.13) capillary glucose checks per week, compared to 11.88 (SD 0.57) checks per week for Hispanic individuals and 10.29 (SD 0.29) checks per week for Black individuals. The mean number of yearly hemoglobin A_1c_ (HbA_1c_) checks was 1.16 (SD 0.15) for Chinese immigrants, 3.31 (SD 0.14) for Hispanic individuals, and 3.23 (SD 0.18) for Black individuals [5].

Diabetes self-management education and counseling programs are effective interventions for diabetes control [11,12]. However, numerous factors limit the access of such programs to Chinese immigrants. While 76.6% of Chinese immigrants report limited English proficiency [13], there is a shortage of language-concordant clinicians and limited language access or medical interpretation [14-17]. Differing cultural norms may limit the relevance and effectiveness of diabetes care and counseling delivered by non-Chinese clinicians or interpreters [6,14,18-20]. Moreover, comprehensive diabetes counseling is impeded by the limited amount of time patients are able to spend with clinicians [15]. As a result, Chinese immigrants often report having unmet information needs for their T2D management [15,17].

Mobile health (mHealth) technology may be a promising way to address some of the previously mentioned barriers and reduce T2D health disparities. Research demonstrates SMS text messaging–based interventions improve glycemic control in patients with T2D, including minority populations [21-24]. SMS text messaging and social media strategies may be particularly relevant for immigrant populations given their high social needs to stay connected with their families and friends in their home countries [25]. To our knowledge, no studies have examined social media–based mHealth interventions in Chinese immigrants with T2D. Little is known regarding access to, use of, and attitudes toward mHealth in this fast-growing immigrant population. While it is often assumed that underserved, low-income immigrant communities have limited access to technology or would not have interest in telehealth programs [26], there is a paucity of empirical data on these subjects. With the rapid growth of telehealth programs over the past few years, it is critical to understand immigrant communities’ access to technology, use of technology for disease management, and interest in future mHealth programs. To address this knowledge gap, this study aims to examine mobile device ownership, social media use, current use of mHealth interventions, and interest in using such technology for T2D management among Chinese immigrants.

## Methods

### Conceptual Framework

This pilot study was informed by the National Institutes of Health (NIH) Stage Model for Behavioral Intervention Development [27] and the National Cancer Institute’s (NCI) Health Information National Trends Survey (HINTS) framework [28]. The NIH Stage Model posits that Stage 0 formative data is critical and can provide important preliminary data to inform Stage I intervention development and evaluation. There is a dearth of culturally tailored interventions to address T2D disparities in low-income older Chinese immigrants. This pilot study examined whether this underserved immigrant population has access to technology and how they access health information. These are critical formative data to collect before allocating resources to developing or testing an intervention.

In addition, we used the HINTS framework [28] to guide the choices of survey questions. Based on the HINTS framework, patients’ health information–seeking behavior is affected by various factors, including patient characteristics (eg, age, gender, socioeconomic status), prior experience with information-seeking, attitudes toward the source, and other contextual factors (eg, access to mobile technologies and Wi-Fi).

### Study Design

For this cross-sectional study, Chinese immigrants with T2D were recruited from 4 community centers in Chinatown areas in NYC. Study flyers were posted in the community centers. Community leaders introduced the study at social events sponsored by the centers, and interested participants self-referred to study staff who attended the events.

To be eligible for the study, participants had to (1) self-identify as a Chinese immigrant or Chinese American, (2) be 18 years of age or older, (3) self-report that they had been diagnosed with T2D over a year ago, and (4) be currently self-managing T2D at home. This study was approved by the New York University Grossman School of Medicine Institutional Review Board. All participants provided signed informed consent. All study materials were available in English, Mandarin, and Cantonese. Surveys were administered face-to-face by bilingual study staff in the participant’s preferred language. Participants received US $20 gift cards as an incentive for completing the survey.

### Measures

#### Sociodemographic Characteristics

Age, gender, marital status, education, income, employment status, duration of residence in the United States, language spoken at home, and English proficiency were assessed.

#### Technology-Related Questions

Measures on technology use were adapted from the HINTS framework, including questions assessing access to technology, current social media use, current use of technology for health management, interest in mHealth interventions, and family or friends’ involvement and interest in mHealth interventions.

#### Access to Technology

Using questions adapted from the HINTS framework, we assessed mobile device ownership (basic cell phones, smartphones, tablets, or none) and access to Wi-Fi at home (yes, no, or don’t know/not sure). For those who owned a smartphone or tablet, we also asked whether they had an unlimited text messaging plan.

#### Current Social Media Use

Current use of social media platforms and SMS text messaging was assessed by asking participants whether they currently used WhatsApp, WeChat (a Chinese version of WhatsApp), basic text messaging, Facebook, or other social media platforms.

#### Current Use of Technology for Health Management

We assessed the extent to which participants relied on technology by asking them to identify their primary source of information for diabetes-related questions. Response options included health care providers; the internet; family, friends, or coworkers; newspapers; and do not seek help. We also asked whether they had used the internet to search for diabetes-related information in the past 12 months and whether they had used mobile phones or computers to watch a health-related video in the past 12 months.

#### Interest in Future mHealth Programs

Participants were asked whether they would be interested in participating in a future mHealth program for T2D self-management.

#### Family and Friends’ Involvement and Interest in mHealth Interventions

Given the importance of family ties in the Chinese culture [6,18,20], we assessed participants’ perceptions of their family’s or friends’ interest in mHealth interventions. Specifically, we asked whether participants discussed their T2D with others and if so, whether these family members or friends would be interested in receiving mHealth interventions to better support the patient in their T2D self-management efforts.

### Statistical Analyses

We recruited 101 participants between April 2018 and July 2018. Data analyses were limited to 91 participants with complete data from the technology use survey. Descriptive statistics were used to examine the distribution of sociodemographic variables in addition to technology ownership and use, whether participants had accessed a health-related video, and attitudes toward mHealth interventions. Means and standard deviations were calculated for continuous variables. Frequencies and percentages were reported for categorical variables. Sample characteristics were summarized. We performed all analyses with SPSS, version 25.0 (IBM Corp).

## Results

As shown in Table 1, the sample was composed of primarily low-income, married, foreign-born, elderly females with limited education, who had long-standing T2D. Most participants reported having lived in the United States for nearly 2 decades and having limited English proficiency.

**Table 1:** Sample characteristics (N=91) and access to technology, current use, and interest in mHealth interventions among older Chinese immigrants with type 2 diabetes.

Characteristic			Value
Age in years, mean (SD)			70 (11)
Age >65 years, n (%)			61 (67)
**Gender, n (%)**			
	Female	57 (63)	
	Male	34 (37)	
Currently married, n (%)			68 (75)
High school education or less, n (%)			58 (64)
**Annual family income, n (%)**			
	≤US $25,000	63 (69)	
	≥US $25,000	12 (13)	
	Declined to answer or don’t know	16 (18)	
**Employment status, n (%)**			
	Currently employed	30 (33)	
	Not employed, not working	9 (10)	
	Retired	52 (57)	
Foreign-born, n (%)			91 (100)
Number of years living in the United States, mean (SD)			19 (14)
Limited English proficiency			78 (86)
**Mobile device ownership, n (%)**			
	Basic mobile phone	20 (22)	
	Smartphone	72 (79)	
	Tablet	49 (54)	
Has a mobile device (basic mobile phone or smart device)			90 (99)
Has a smart device (smartphone or tablet)			83 (91)
**Social media** **platforms (including text messaging) currently used by participant** **, n (%)**			
	WeChat	65 (71)	
	Basic SMS text messaging via cellular carrier	62 (68)	
	WhatsApp	4 (4)	
Has Wi-Fi installed in home, n (%)			66 (73)
**Has unlimited SMS text messaging plan, n (%)**			
	Yes	36 (40)	
	No	41 (45)	
	Don’t know	13 (14)	
	Did not answer	1 (1)	
**Primary source of diabetes-related information, n (%)**			
	Health care provider	63 (69)	
	The internet	13 (14)	
	Family, friends, or coworkers	10 (11)	
	Newspapers	4 (4)	
	Do not seek help	1 (1)	
Has used the internet to look for information about diabetes in the past 12 months, n (%)			21 (23)
Has watched a health-related video in the past 12 months, n (%)			34 (37)
Is interested in receiving mHealth interventions for T2D, n (%)			69 (76)
Has family/friends to talk to about their T2D, n (%)			62 (68)
Family/friends interested in receiving mHealth interventions to better support participant^a^, n (%)			50 (81)

^a^This question was only assessed as a follow-up item among 62 participants who reported that they had family or friends to talk to about their T2D. The percentage was calculated accordingly.

Nearly all participants had a smart mobile device, and nearly three-quarters had Wi-Fi access at home. The most commonly used social media platform was WeChat, followed by basic text messaging. Very few participants used WhatsApp, and none reported using Facebook, Twitter, or Instagram.

The majority reported that their primary source for diabetes-related information is health care clinicians, with few participants reporting their first sources of information are the internet, family, friends, or coworkers. Less than one-quarter of the sample reported using the internet to search for diabetes-related information in the past 12 months. About one-third of the sample reported that they had watched a health-related video on their cell phone or computer in the past 12 months.

Despite the fact that the majority of participants were low-income older immigrants with limited education, over three-quarters expressed interest in receiving T2D management mHealth interventions in the future. About two-thirds reported having family or friends to talk to about their T2D. Of these, a large majority agreed that family or friends would be interested in receiving mHealth interventions to learn how to best support them in their efforts to manage their T2D.

## Discussion

### Principal Findings

To the best of our knowledge, this is the first study that provides data on technology ownership, current use, and interest in mHealth interventions in underserved Chinese immigrants with T2D. Although the majority of study participants were older immigrants with low income and limited education, they demonstrated high mobile device ownership and familiarity with a particular social media platform (ie, WeChat). This finding is consistent with a previous study reporting high social media use among immigrant populations to connect with their families and friends in their home countries [25]. Our data are also consistent with recent survey results from the Pew Research Center showing that about 95% of White, Hispanic, and Black American adults owned a cell phone and 80% had a smartphone [29]. The wide availability of mobile devices suggests a promising platform to increase access and deliver diabetes messaging and support to this underserved population [30]. Of particular note, Chinese immigrants are familiar with WeChat, a popular free Chinese social media app, and rarely used WhatsApp, Facebook, or Twitter. These data suggest that researchers may want to consider leveraging WeChat for this population in the future.

Despite high smart device ownership and internet access, the use of technology for health-related purposes was low in our sample. While about 70% of US adults considered the internet as their top source for health information [31], only 14% (13/91) of participants in our sample did, with the majority relying on health care providers. Compared to about 80% of US adults using the internet to search for health-related information [32], less than one-quarter of our sample (21/91, 23%) did so in the past 12 months. One interesting finding to note is that while the use of the internet to search for health-related information was much lower in our sample than the general US population, the proportion of participants who had watched a health-related video in the past 12 months was comparable [33]. This may be related to the high use of WeChat in our sample, which permits sharing of videos via chat windows. Our study found that almost 80% (69/91, 76%) of this relatively elderly sample reported interest in receiving mHealth videos in the future to help with T2D management. Taken together, these data suggest future researchers may want to consider leveraging multimedia strategies (eg, brief videos) to increase access and uptake of T2D-related education and counseling in this population.

The success of T2D management depends largely on the social and environmental contexts in which patients live and perform diabetes self-care [34,35]. When patients with T2D live in a supportive family context (eg, the family embraces a lifestyle consistent with the needs of the patient with T2D), they are more likely to receive social and emotional support for self-management, adhere to diabetes self-care requirements, and achieve better glycemic control [36]. Patients with T2D living in nonsupportive family environments (eg, family members undermine the patient’s efforts) have more difficulty initiating and sustaining recommended diabetes self-care behaviors, experience more diabetes-related distress, and report lower self-efficacy for self-management success [36,37]. Involving family members may be a useful strategy, given our finding that most participants discussed their T2D with family and friends, and believed their family and friends would be interested in receiving mHealth-based guidance to better support the participant’s self-management efforts.

Our findings provide valuable implications in the era of the COVID-19 pandemic. It is well-documented that COVID-19 disproportionately affects racial and ethnic minorities and immigrant communities [38-40]. The social isolation and loneliness resulting from prolonged safety measures (eg, social distancing, stay-at-home orders) is particularly concerning for older individuals and those with T2D who are at high risk of poor COVID-19 outcomes [41]. A high rate of mobile device ownership and strong interest in mHealth interventions among older Chinese immigrants with T2D may represent a window of opportunity for low-cost mHealth interventions to reduce diabetes burden and improve self-management outcomes in this rapidly growing immigrant group in the era of COVID-19.

### Limitations and Strengths

There are several limitations to be acknowledged. This study involved a relatively small, convenient sample. Participants were recruited from the NYC metropolitan area, limiting generalizability to other locations. Because recruitment was accomplished through community-based organizations, the results may not be representative of all Chinese Americans receiving care through primary care or hospital settings. Participants in this study had, on average, lived in the United States for almost 20 years. These results may not be applicable to newly arrived immigrants who might be less acculturated or less familiar with western approaches for T2D management.

Several strengths should also be noted. To the best of our knowledge, this is the first study that provides data on technology use in older low-income Chinese immigrants with T2D. These data are particularly relevant during the COVID-19 pandemic. Our data on the high usage of WeChat and basic text messages and rare usage of WhatsApp suggested future researchers may consider choosing linguistically and culturally tailored platforms to engage racial and ethnic minorities. In addition, Chinese immigrants have been reported to be a hard-to-reach population for research participation [42,43]. We partnered with several trusted community organizations and successfully recruited 101 Chinese immigrants within 4 months (April 2018 to July 2018).

This study is important in that it focuses on a fast-growing, yet significantly understudied immigrant population [44]. While telehealth and telemedicine programs are rapidly growing in the era of the COVID-19 pandemic, low-income, underserved populations, particularly older immigrants with limited English proficiency, are often overlooked because it is assumed that they do not have access to technology and have limited interest in telehealth programs [26]. This study serves as a first step to dispel these myths and demonstrates that access to technology is not a major issue and that interest in mHealth programs is high. Our research team is currently testing a WeChat-based culturally and linguistically tailored diabetes video program in this underserved, older, low-income immigrant population.

### Conclusions

This study addressed a critical gap in the literature with regard to technology ownership and use of and attitudes toward mHealth in a relatively older immigrant population with T2D. While current telehealth use remained low, the high mobile device ownership and social media use and strong interest in mHealth programs suggests that mHealth may be a promising approach to deliver health education and increase access to diabetes support to this fast-growing minority group. This study also provides timely preliminary data in the era of COVID-19 as older immigrant communities are among the hardest hit populations. With the rapid shift to telemedicine strategies, it is important for health care policymakers, clinicians, community partners, and researchers to consider how to leverage existing technologies to reduce health disparities and increase access to health care in this underserved population.

## Abbreviations

HbA_1c_: hemoglobin A_1c_
HINTS: Health Information National Trends Survey
mHealth: mobile health
NCI: National Cancer Institute
NIH: National Institutes of Health
T2D: type 2 diabetes
